# Integration of transcriptomic and proteomic analyses for finger millet [*Eleusine coracana* (L.) Gaertn.] in response to drought stress

**DOI:** 10.1371/journal.pone.0247181

**Published:** 2021-02-17

**Authors:** Jiguang Li, Yanlan Wang, Liqun Wang, Jianyu Zhu, Jing Deng, Rui Tang, Guanghui Chen

**Affiliations:** 1 Agricultural College, Hunan Agricultural University, Changsha, Hunan, China; 2 Crop Research Institute, Hunan Academy of Agricultural Sciences, Changsha, Hunan, China; East Carolina University, UNITED STATES

## Abstract

Drought is one of the most significant abiotic stresses that affects the growth and productivity of crops worldwide. Finger millet [*Eleusine coracana* (L.) Gaertn.] is a C_4_ crop with high nutritional value and drought tolerance. However, the drought stress tolerance genetic mechanism of finger millet is largely unknown. In this study, transcriptomic (RNA-seq) and proteomic (iTRAQ) technologies were combined to investigate the finger millet samples treated with drought at different stages to determine drought response mechanism. A total of 80,602 differentially expressed genes (DEGs) and 3,009 differentially expressed proteins (DEPs) were identified in the transcriptomic and proteomic levels, respectively. An integrated analysis, which combined transcriptome and proteome data, revealed the presence of 1,305 DEPs were matched with the corresponding DEGs (named associated DEGs-DEPs) when comparing the control to samples which were treated with 19 days of drought (N1-N2 comparison group), 1,093 DEGs-DEPs between control and samples which underwent rehydration treatment for 36 hours (N1-N3 comparison group) and 607 DEGs-DEPs between samples which were treated with drought for 19 days and samples which underwent rehydration treatment for 36 hours (N2-N3 comparison group). Gene ontology (GO) and Kyoto Encyclopedia of Genes and Genomes (KEGG) analysis identified 80 DEGs-DEPs in the N1-N2 comparison group, 49 DEGs-DEPs in the N1-N3 comparison group, and 59 DEGs-DEPs in the N2-N3 comparison group, which were associated with drought stress. The DEGs-DEPs which were drought tolerance-related were enriched in hydrolase activity, glycosyl bond formation, oxidoreductase activity, carbohydrate binding and biosynthesis of unsaturated fatty acids. Co-expression network analysis revealed two candidate DEGs-DEPs which were found to be centrally involved in drought stress response. These results suggested that the coordination of the DEGs-DEPs was essential to the enhanced drought tolerance response in the finger millet.

## Introduction

Finger Millet [*Eleusine coracana* (L.) Gaertn.] (2n = 4x = 36, AABB) belongs to the Poaceae family and is the most widely grown cereal crop after pearl millet and foxtail millet [1]. It is widely cultivated in arid and semi-arid tropical regions of the world, such as Africa and South Asia. The cultivation area of finger millet globally represents about 3.38 million hectares and the production of grain is about 3.76 million tons annually (FAOSTAT database, 2007, http://www.faostat.fao.org). Compared with many other grains, finger millet is more rich in high-quality proteins (5–8%), aromatic compounds, tryptophan, cysteine, methionine, dietary fiber (15–20%), phytochemicals, calcium (2–3%), manganese, iron, phosphorus, copper, sodium and a large variety of carbohydrates. It is gluten-free and known for its health benefits [2–4]. In addition to its potential to solve some nutritional deficiencies in rural areas, finger millet is a typical C_4_ plant that copes well with drought conditions as well as biotic stresses [5].

Climate change represents a significant threat to the sustainable development of agriculture, with increasing drought stress likely leading to a significant impairment of crop growth [6]. Due to global climate change caused by the greenhouse effect, droughts have become more severe and frequent. Drought affects various physiological and biochemical processes involved in plant growth and development, including photosynthesis, ion absorption and transport, chlorophyll synthesis, respiration and carbohydrate metabolism, thereby inhibiting plant growth and reducing yield [7]. During drought stress, plants will respond with various different self-protection mechanisms. It has previously been shown that there are a series of changes in enzyme activity, cell penetration materials, metabolites, endogenous hormones and reactive oxygen species to deal with drought and dehydration [8]. Additionally, drought tolerance is increased through the regulation of ABA- and ROS- mediated signaling. For example, the *AtERF71/HRE2* transcription factor has been shown to mediate the ABA signal pathway of drought stress response as well as hypoxia response in *Arabidopsis* [9]. Plants also adept to drought stress by inducing the expression of specific drought tolerance genes. Many such genes, such as the classic calcineurin B1 (*CBL1*) gene, are conserved across multiple species, which plays a key role as a positive regulator of salt and drought response in *Arabidopsis* [10]. Additionally, *ABO3*, a WRKY transcription factor, mediates plant responses to drought tolerance in *Arabidopsis* [11]. A significant amount of research has been conducted on the drought tolerance of finger millet, including studies focused on variations in drought tolerance components and their association with yield in finger millet. It has also been shown that higher water use contributes more to shoot biomass, while higher transpiration efficiency contributes more to grain yield [12]. Meanwhile, the characterization of physiological and biochemical responses of finger millet varieties during drought stress has also been studied, providing an important basis for selecting drought-tolerant varieties [13]. However, the drought tolerance mechanisms at the level of individual genes and proteins are still unclear. Effective improvement in drought tolerance requires a thorough understanding of gene expression regulation and protein interaction mechanisms under drought stress.

The past decade has seen the rapid development and popularization of high-throughput sequencing technology which has greatly increased knowledge about the relationship between genotype and phenotype. RNA-seq transcriptome analysis has been widely used in plant genetics research, revealing DEGs in various biological processes. For example, genome-wide association mapping for seed protein content in a global finger millet collection was carried out through genotyping by sequencing [14]. Meanwhile, improvements in high-throughput liquid chromatography-tandem mass spectrometry (LC-MS/MS) have made proteomics a powerful tool. Study of protein expression levels, post-translational modifications and protein-protein interactions have led to a more comprehensive understanding of cell metabolism and other processes which occur at the protein level. At present, proteomic analysis has been performed extensively in many plant species during drought stress to reveal protein expression and interaction effects, including research in rice [15] and wheat [16].

In this work, a transcriptome and proteome sequencing were carried out in finger millet under normal growth conditions, 19 days drought-stressed conditions, and 36 hours rehydration conditions. The possible biological functions of the resulting DEPs and DEGs were assessed, the interaction and expression relationships between these DEPs and DEGs were explored, and their potential effects on millet drought stress were assessed. In all, 113 DEGs-DEPs have different levels of expression during drought stress, and network analysis indicated that two DEGs-DEPs play a critical regulatory role. This study provides a reference for further understanding finger millet regulatory networks under drought stress, and represents an essential genetic resource for finger millet drought-tolerant germplasm innovation.

## Materials and methods

### Plant materials and drought treatment

Finger millet [*Eleusine coracana* (L.) Gaertn.] was used as the experimental material in this study. Seeds were planted in the Hunan Academy of Agricultural Sciences, Changsha, China (113°4 ’50 "E, 28°12’ 15" N). Seeds were seeded in a growth chamber and cultivated to the four true leaf stage. Afterwards, the seedlings were transplanted into a 15 cm×23 cm nursery pot containing a nutrient substrate for routine management. At the six-leaf stage, uniform and healthy seedlings were selected and cultivated under a 16h (light) / 8h (dark) cycle at 28°C (light) / 24°C (dark), with a photosynthetic photon flux density of 300 μmol/m^2^/s^1^ and a relative humidity 65%-70% in a controlled environment chamber. After 15 days of normal cultivation, natural drought treatment was given. The drought sample was grown for 19 days without water and termed N2. N3 was sampled after 36 hours of rehydration, following 19 days of drought. The control sample on day 34 of normal growth was called N1. After collection, all samples were immediately frozen in liquid nitrogen and stored at -80°C.

A plant malondialdehyde (MDA) assay kit (A003-1, TBA thiobarbituric acid method), plant total superoxide dismutase (T-SOD) assay kit (A001-1, hydroxylamine method), plant peroxidase (POD) assay kit (A084-3), plant catalase (CAT) assay kit (A007-1-1, visible spectrophotometry method), soluble sugar assay kit (A145-1-1, anthrone colorimetry method), glutathione reductase (GR) assay kit (A062-1-1) and hydroxyl radical kit (A018-1-1, colorimetry method) were used to determine the MDA, SOD, POD CAT, soluble sugar, GR and hydroxyl radical content, respectively (Nanjing Jiancheng Biotechnology Co., Ltd). A root activity kit (TP1013, Naphthylamine colorimetry method) (Beijing Leagene Biotechnology Co., Ltd) and Proline (PRO) kit (BC0290, visible spectrophotometry method) (Beijing Solarbio Science & Technology Co., Ltd) were used to detect root activity and PRO content.

### Transcriptome analysis

Total RNA extraction of finger millet leaf tissue was conducted using a TransZol kit (TransGen Biotech, Inc., Beijing, China). Sequencing libraries were constructed using an NEBNext^®^ Ultra^™^RNA Library Prep Kit for Illumina^®^ (NEB, Ipswich, MA, USA). There were three biological replicates in the N1, N2 and N3 group. Paired-end non-strand specific libraries (200-250bp) were constructed and sequenced based on the Illumina HiSeq^™^ X-Ten platform (BGI, Shenzhen, China). FastQC was used to control the quality of sequencing data [17] and Trimmomatic-0.36 was used to remove low-quality sequences [18]. Bowtie2 aligns clean reads to the genome sequence [19], followed by the use of RSEM to calculate the expression level of each sample [20]. The resulting quantifications were expressed as fragments per kilobase million (FPKM) [21]. The differentially expressed genes (DEGs) were screened using DEseq2 based on an absolute fold change value of |log_2_foldchange| > 1 and an adjusted *P* value ≤ 0.01 [22].

### Proteome analysis

Samples of finger millet leaves tissue were sonicated three times on ice using a high intensity ultrasonic processor (Scientz) in lysis buffer (8 M urea, 1% Protease Inhibitor Cocktail). The remaining debris was removed by centrifugation at 12,000 g at 4 °C for 10 min. Finally, the supernatant was collected and the protein concentration was determined with a BCA kit (Beyotime Biotechnology P0011-1 Shanghai China). Peptides were desalted by a Strata X C18 SPE column (Phenomenex) and vacuum-dried. Peptides were reconstituted in 0.5 M TEAB and processed according to the TMT kit/iTRAQ kit instructions ([10 plex] ThermoFisher Scientific 90406 Waltham USA). Briefly, one unit of TMT/iTRAQ reagent was thawed and reconstituted in acetonitrile. The peptide mixtures were then incubated for 2 h at room temperature and pooled, desalted and dried by vacuum centrifugation. The tryptic peptides were fractionated by high pH reverse-phase HPLC using a Thermo Betasil C18 column (5 μm particles, 10 mm ID, 250 mm length). Peptides were first separated with a gradient of 8% to 32% acetonitrile (pH = 9.0) over 60 min into 60 fractions. Then, the peptides were combined into 6 fractions and dried by vacuum centrifugation.

The tryptic peptides were dissolved in 0.1% formic acid (solvent A), then directly loaded onto a home-made reversed-phase analytical column (15-cm length, 75 μm i.d.). The gradient was 80% for the last 3 min, at a constant flow rate of 400 nL/min on an EASY-nLC 1000 UPLC system. The peptides were subjected to an NSI source followed by tandem mass spectrometry (MS/MS) in a Q ExactiveTM Plus (Thermo) coupled to the UPLC. The electrospray voltage applied was 2.0 kV. The m/z scan range was 350 to 1800 for a full scan, and intact peptides were detected in the Orbitrap at a resolution of 70,000. Peptides were then selected for MS/MS using an NCE setting of 28 and the fragments were detected in the Orbitrap at a resolution of 17,500. The resulting MS/MS data were processed using the Maxquant search engine (v.1.5.2.8). Proteins with fold-change > 1.3 relative to the control, and FDR-corrected *P*-values < 0.05, were considered to be significantly different.

### Data and statistical analysis

A Gene Ontology (GO) enrichment analysis was performed using singular enrichment analysis in the agriGO online tool [23]. GO annotation of the proteome was derived from the UniProt-GOA database (http://www.ebi.ac.uk/GOA/). Protein domain functional descriptions were annotated by InterProScan (http://www.ebi.ac.uk/interpro/) based on the protein sequence alignment method, and the InterPro domain database was used. Gene functions were annotated based on the Clusters of Orthologous Groups of proteins (KOG/COG) database (http://www.ncbi.nlm.nih.gov/COG). The protein sequences were submitted to the Encyclopedia of Genes and Genomes (KEGG) online service tool KAAS (v.2.0, http://www.genome.jp/kaas-bin/kaas_main) to annotate the KEGG database description. The annotation result was then mapped onto the KEGG pathway database using the KEGG online service tool KEGG mapper. Wolfpsort online subcellular localization predication software (https://www.genscript.com/wolf-psort.html) was used to predict subcellular localization of proteins.

All differentially expressed protein database accessions or sequences were searched against the STRING database version 11.0 for protein-protein interactions, with all interactions possessing a confidence score ≥ 0.7 (high confidence) retained. The resulting interaction network from STRING was visualized in the R package “networkD3”. Cytoscape was used to generate a co-expression plot. A graph oretical clustering algorithm, molecular complex detection (MCODE), was utilized to analyze densely connected regions. MCODE is part of the plug-in tool kit of the network analysis and visualization software Cytoscape [24].

## Results

### Drought treatment and determination of physiological indicators

**Fig 1:**
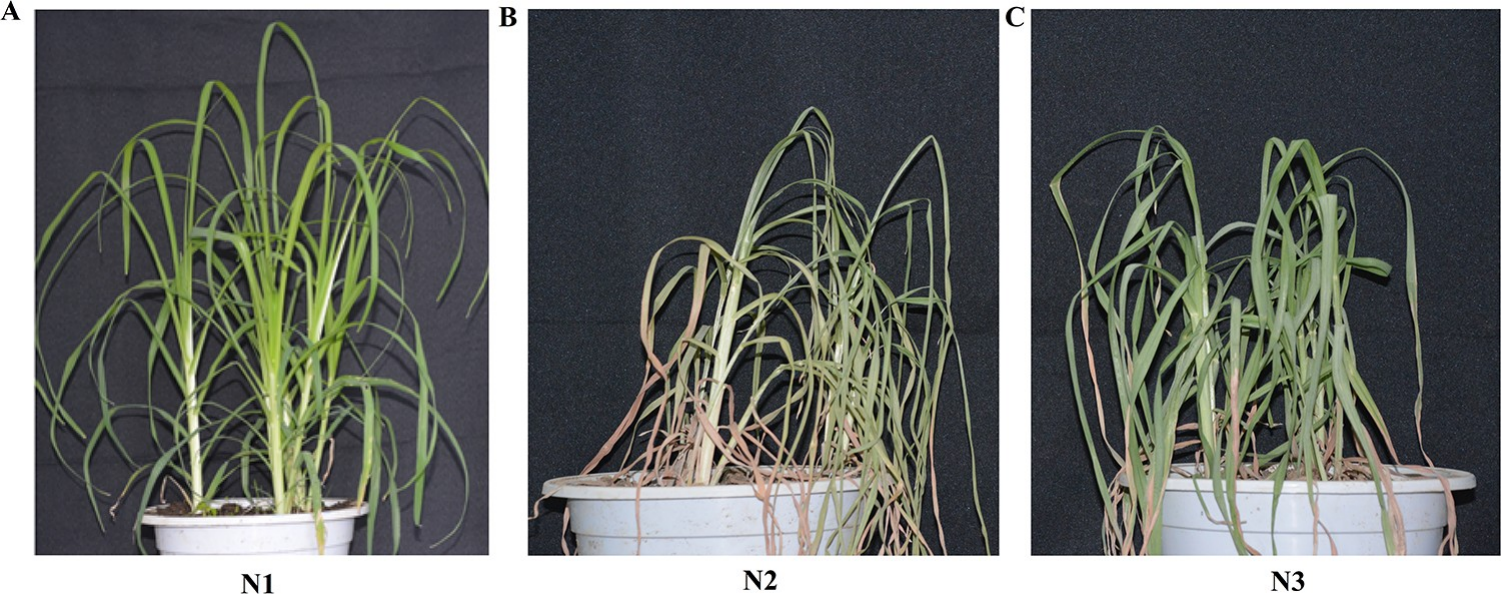
Phenotyping of finger millet at different stages. (A) Control plants (N1). (B) Plants after 19 days of drought stress (N2). (C) Plants which were rehydrated for 36 hours after drought stress (N3).

**Fig 2:**
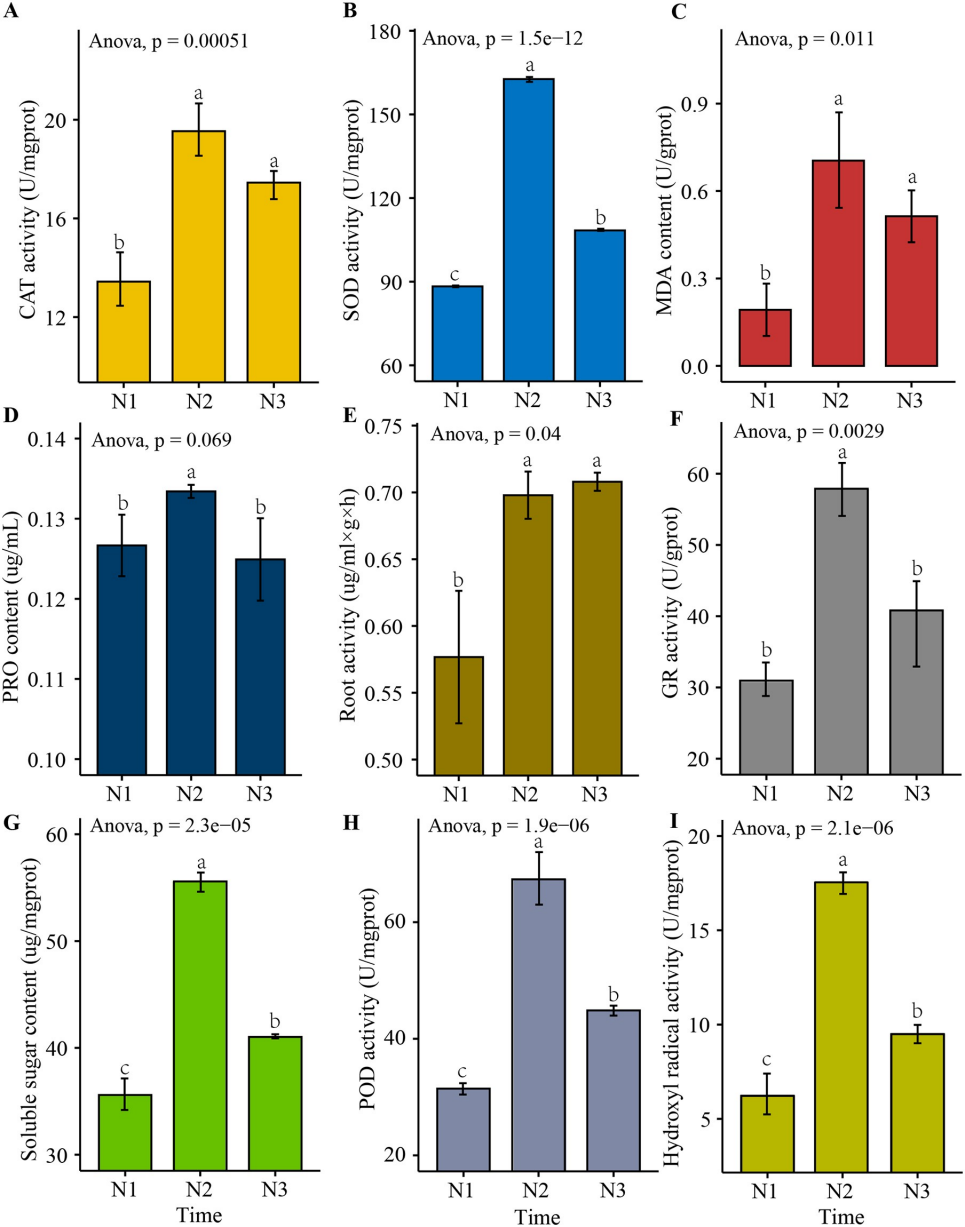
The activities of CAT (A), SOD (B), PRO (D), GR (F), POD (H), hydroxyl radical (I), MDA (C) soluble sugar contents(G) and root activity(E) in finger millet leaves under control(N1), after 19 days of drought (N2) and rehydration treatment for 36 hours (N3) treatments.

Six-leaf finger millet was subjected to drought treatment. Plants grown under normal conditions were used as controls (N1). After 19 days of drought treatment (N2), rehydration treatment for 36 hours was used to generate the N3 treatment. Phenotypic examination revealed that the 19-day drought-stressed plants were severely wilted, but still viable. The plants which were rehydrated for 36 hours after drought stress recovered significantly, indicating a strong degree of drought tolerance (Fig 1). In addition to visual inspection, protective enzyme activities, membrane permeable root activities, soluble sugar content and hydroxyl radical activity were measured. Under the same growth conditions and different drought periods, physiological and biochemical adaptations, such as significant increases in the level of SOD (superoxide dismutase), POD (peroxidase), CAT (catalase), GR (Glutathione reductase), PRO (Proline) and hydroxyl radicals, were seen at 19 days of drought stress. These compounds decreased by 36 hours of rehydration after drought. MDA (Malondialdehyde) content and soluble sugar content directly reflect the degree of peroxidation of plant cell membranes. Compared with the control, the content of these markers increased significantly after 19 days of drought, and the activity decreased after 36 hours of rehydration. Additionally, these markers increased in root during drought stress and continued to increase after 36 hours of rehydration (Fig 2). Taken together, these physiological indicators point to a strong drought tolerance phenotype for finger millet.

### Transcriptomic analysis

RNA-seq was performed using the different drought stress conditions of finger millet, and each stage included three biological replicates. After removing the adaptor and low-quality sequence, RNA-seq received 94,844 transcripts. There are 28,733 DEGs between N1 and N2, 22,175 DEGs between N1 and N3 and 29,694 DEGs between N2 and N3 were identified (|log2 FC| ≥ 1, FDR ≤ 0.01). Among them, 13,930 were up-regulated and 14,803 were down-regulated between N1 and N2, 11,246 were up-regulated and 10,929 were down-regulated between N1 and N3 and 15,260 were up-regulated and 14,434 were down-regulated between N2 and N3 (S1A Fig). In total, 8240 DEGs were identified in all comparison groups, 4,883, 2,340 and 4,411 genes accumulated specifically in the group N1-N2, N1-N3 and N2-N3 comparisons, respectively (S1B Fig).

**Fig 3:**
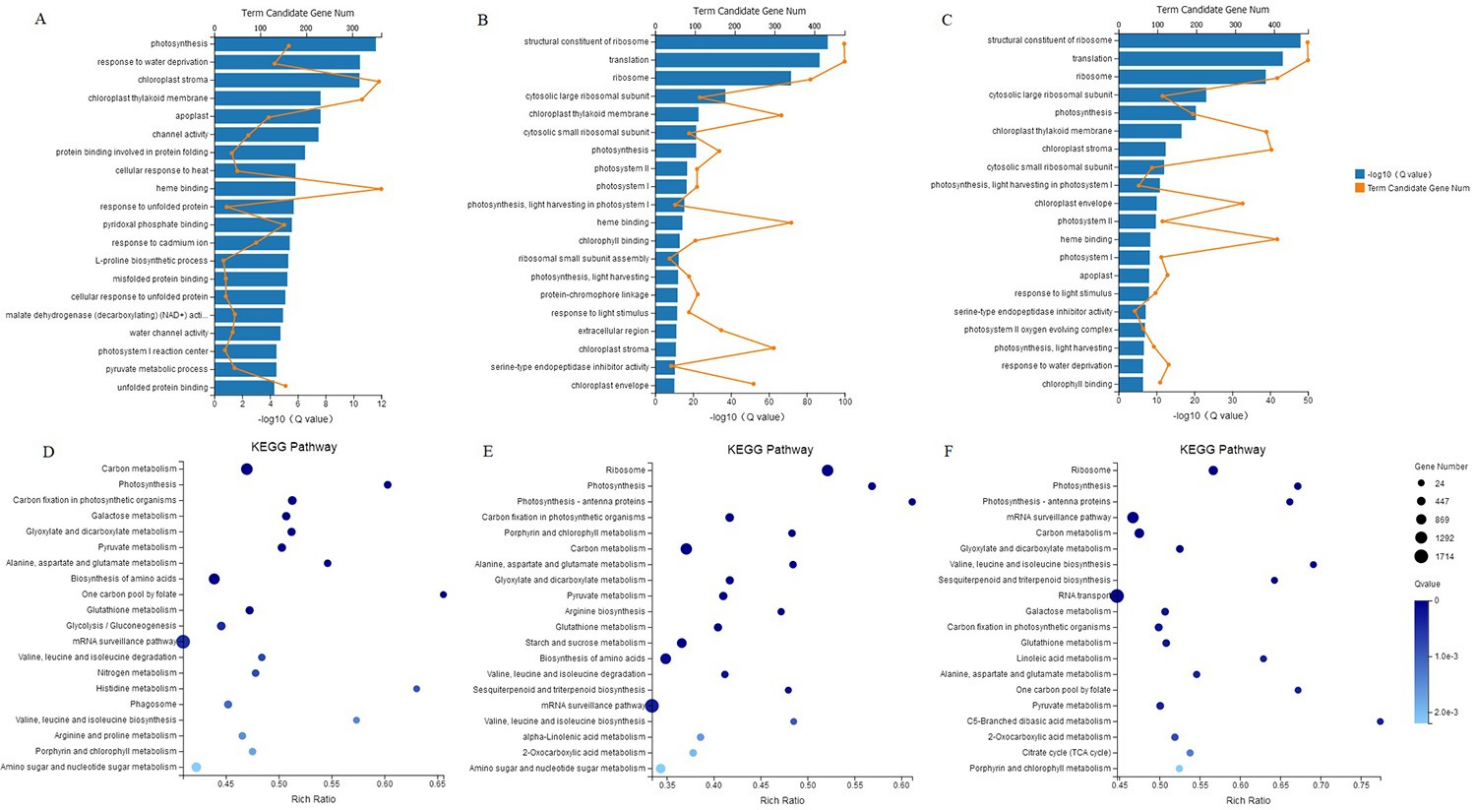
GO and KEGG pathway enrichment analysis of the DEGs of different drought treatment comparison groups of finger millet. (A) GO enrichment of the N1-N2 DEGs group. (B) GO enrichment of the N1-N3 DEGs group. (C) GO enrichment of the N2-N3 DEGs group. (D) KEGG pathways of the N1-N2 DEGs group. (E) KEGG pathways of the N1-N3 DEGs group. (F) KEGG pathways of the N2-N3 DEGs group.

GO enrichment analysis of different drought treatment comparison groups of finger millet DEGs is shown in Fig 3A–3C. DEGs of the N1-N2 comparison group were highly enriched in photosynthesis, response to water deprivation and chloroplast stoma. DEGs of N1-N3 and N2-N3 comparison groups were highly enriched in structural constituent of ribosome, translation and ribosome process. KEGG pathway analysis revealed that the N1-N2 comparison group DEGs were significantly enriched in mRNA surveillance pathway and carbon metabolism. The N1-N3 comparison group DEGs were highly enriched in mRNA surveillance pathway and ribosome, while the N2-N3 comparison group DEGs were highly enriched in RNA transport and mRNA surveillance pathway (Fig 3D–3F).

### Quantitative proteomics analysis

In the quantitative proteomics analysis, 72,823 spectra were generated using label-free analysis in a finger millet fertile line. The spectrum utilization rate was 27.0% and a total of 39,826 peptides were identified, 28,316 of which were unique. There were 7,367 protein species identified, of which 5,587 could be quantified (FDR ≤ 0.01) (S1C Fig). Based on the threshold for screening DEPs (|log2 FC| ≥ 1.3, p ≤ 0.05), there were 808 up-regulated and 497 down-regulated DEPs in the N1-N2 comparison group as well as 737 up-regulated and 356 down-regulated peptides in the N1-N3 comparison group. There were also 348 up-regulated and 259 down-regulated peptides in the N2-N3 comparison group (S1D Fig). Subcellular localization prediction indicated that 3,292 DEPs were localized to the chloroplast, with 600 proteins (45.98% of all DEPs in the N1-N2 group) in the N1-N2 comparison group, 516 proteins (47.21% of all DEPs in the N1-N3 group) in the N1-N3 comparison group, and 287 proteins (47.28% of all DEPs in the N2-N3 group) in the N2-N3 comparison group. There were 1516 DEPs predicted to be localized to the cytoplasm, and 1274 DEPs predicted to be localized to the nucleus (S2D–S2F Fig). These results indicate that these DEPs could form a complex regulatory network for drought tolerance in finger millet.

**Fig 4:**
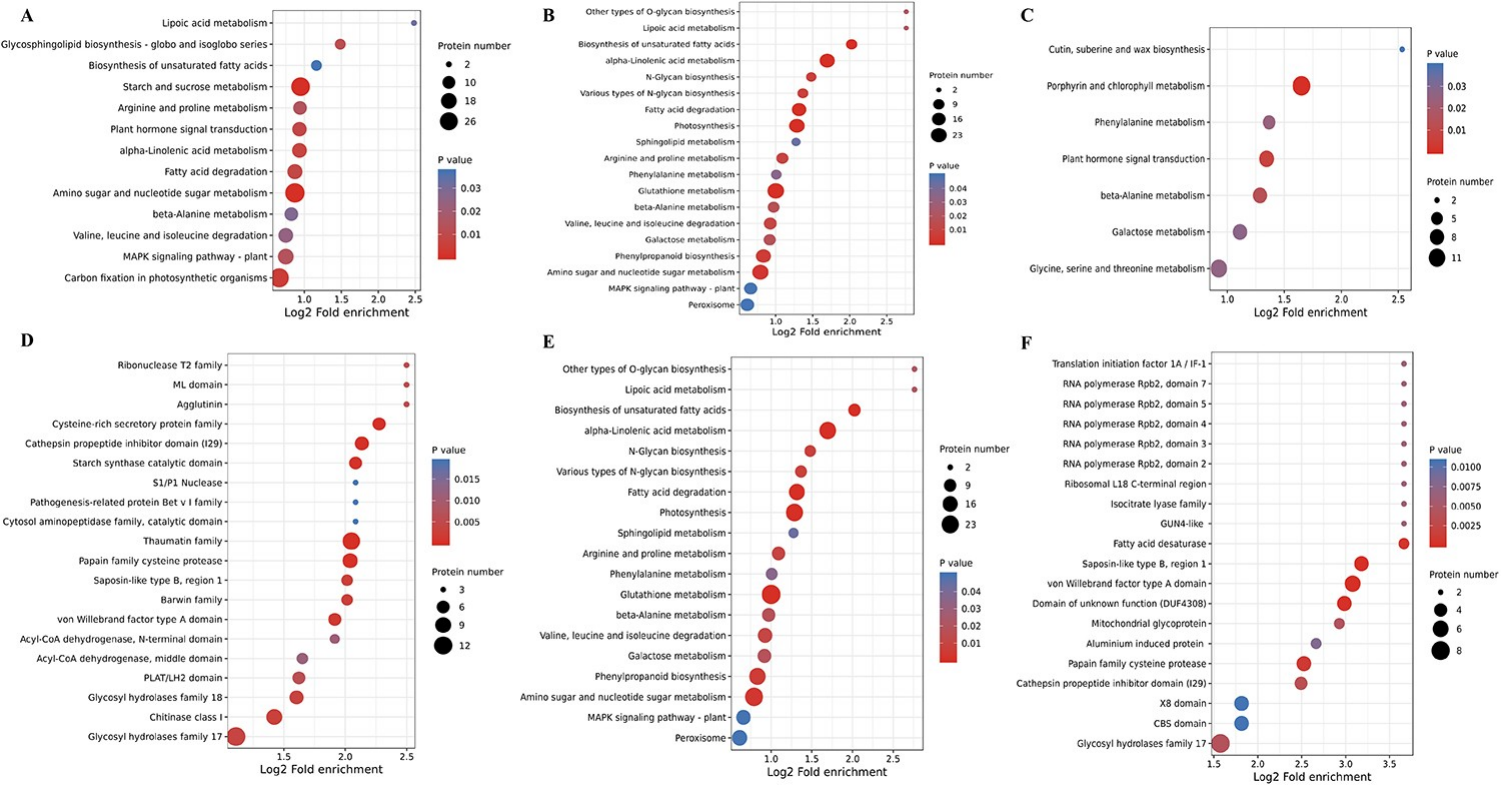
KEGG pathway and domain prediction analysis of the DEGs of different drought treatment comparison groups. (A) KEGG pathways of the N1-N2 DEPs group. (B) KEGG pathways of the N1-N3 DEPs group. (C) KEGG pathways of the N2-N3 DEPs group. (D) Domain prediction analysis of the N1-N2 group. (E) Domain prediction analysis of the N1-N3 group. (F) Domain prediction analysis of the N2-N3 group.

GO enrichment analysis of different drought treatment comparison groups of finger millet indicated that the DEPs were highly enriched in cellular metabolic process, organic substance metabolic process and primary metabolic process in the biological process category (S3 Fig). Intracellular, intracellular organelle and membrane-bounded organelle were enriched in the cellular component category. Organic cyclic compound binding, heterocyclic compound binding and hydrolase activity were enriched in the molecular function category. The functions of the DEPs were then classified in the COG/KOG database. It was found that DEPs during drought stress were enriched for posttranslational modification, protein turnover and chaperones. The N1-N2, N1-N3, N2-N3 comparison groups had 115, 102 and 57 DEPs in this functional category, respectively (S2A–S2C Fig). KEGG pathway analysis revealed that the DEPs of the N1-N2 comparison group under drought stress were significantly enriched in starch and sucrose metabolism, amino sugar and nucleotide sugar metabolism and carbon fixation in photosynthetic organisms. The KEGG pathways of DEPs in the N1-N3 comparison group were highly enriched in alpha–linolenic acid metabolism, photosynthesis, glutathione metabolism and amino sugar and nucleotide sugar metabolism. DEPs in the N2-N3 comparison group were enriched in porphyrin and chlorophyll metabolism, glycine, serine and threonine metabolism as well as glycine, serine and threonine metabolism pathways (Fig 4A–4C). Additionally, DEPs of the N1-N2 comparison group were significantly enriched in glycosyl hydrolases family 17, thaumatin family and chitinase class I domains. The N1-N3 comparison group DEPs domain annotations largely were glutathione S#x2013;transferase, N#x2013;terminal domain, glycosyl hydrolases family 17, thaumatin family and peroxidase. The N2-N3 comparison group DEPs domain annotations mostly were glycosyl hydrolases family 17 and von Willebrand factor type A domain (Fig 4D–4F).

### Correlations analysis between transcriptome and proteome data

An integrated analysis of the proteomic and transcriptomic data produced in this study was conducted to determine the correlation between the two different data types. In total, 94,844 transcripts and 7,367 proteins were identified and quantified in the transcriptome and proteome analyses, respectively, and 7,290 genes were detected in both analyses. Additionally, there were 28,733 DEGs and 1,309 DEPs between control plants and plants treated with 19 days of drought, 22,175 DEGs and 1,093 DEPs between plants treated with 19 days of drought and plants which were subjected to rehydration for 36 hours after drought and 29,694 DEGs and 607 DEPs between control plants and plants which were rehydrated for 36 hours (S1E Fig).

**Fig 5:**
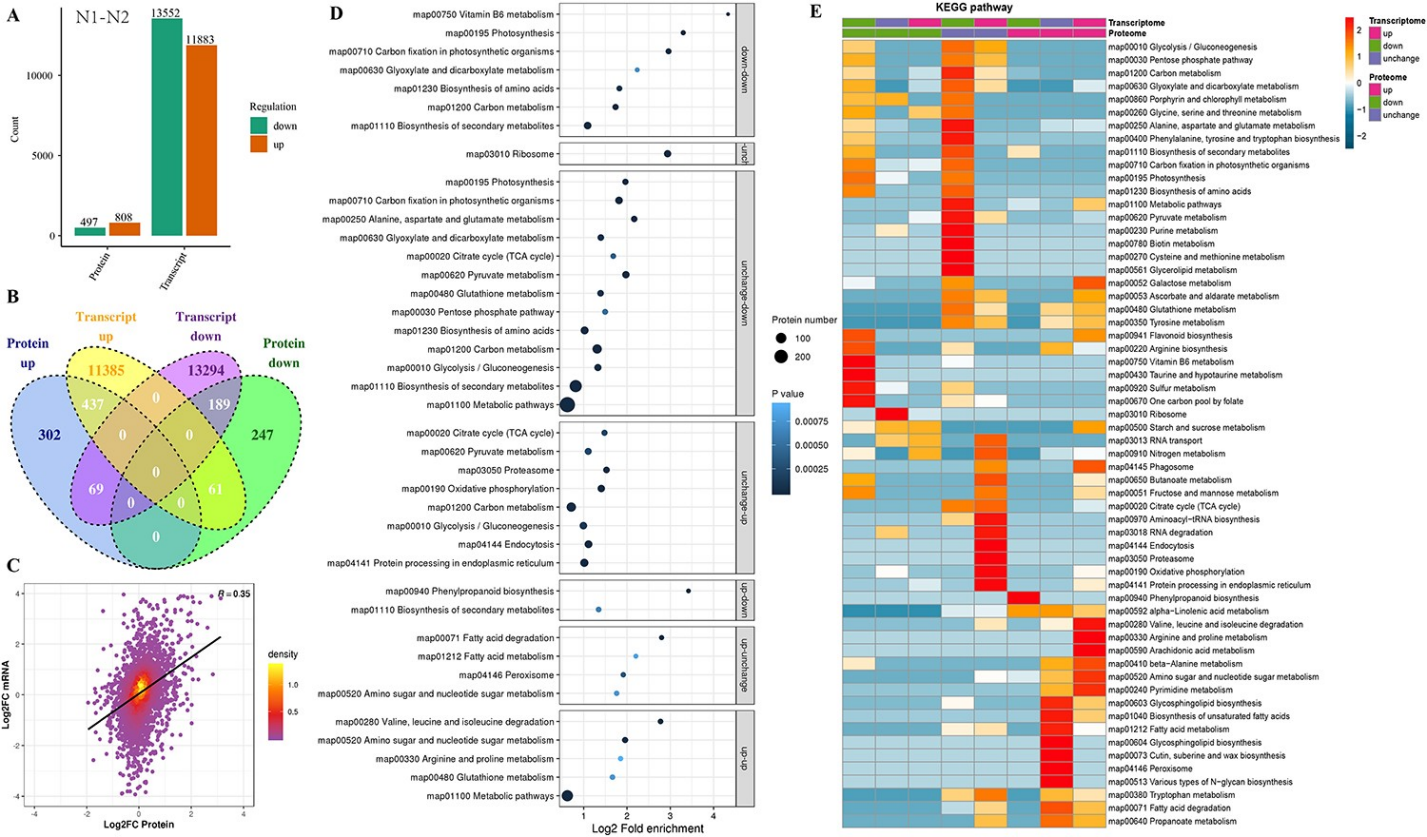
(A) DEGs-DEPs distribution statistics in the N1-N2 DEPs group. (B) Venn diagram showing comparative analysis of DEGs-DEPs in the N1-N2 DEPs group. (C) Scatter plot between a given transcript and its one-to-one corresponding protein expression in the N1-N2 DEPs group. (D) GO enrichment analysis of the N1-N2 comparison group of DEGs-DEPs. (E) KEGG pathway analysis of the N1-N2 comparison group of DEGs-DEPs.

Compared with N1-N2 group in Fig 5A and 5B, there are a total of 800 DEPs are up-regulated and 492 are down-regulated 1,955 DEGs are up-regulated and 1,244 are down-regulated. A total of 294 DEPs are up-regulated and matched with their corresponding DEGs are unchanged (DEPs matched with their corresponding DEGs named associated DEGs-DEPs), 242 DEPs are down-regulated and matched with their corresponding DEGs are unchanged, 1,457 DEPs are unchanged and matched with their corresponding DEGs are up-regulated, 986 DEPs are unchanged and matched with their corresponding DEGs are down-regulated. Meanwhile, 189 DEPs and their corresponding DEGs are both down-regulated, 437 DEPs and their corresponding DEGs are both up-regulated, 61 DEPs are down-regulated and their corresponding DEGs are up-regulated, 69 DEPs are up-regulated and their corresponding DEGs are down-regulated, respectively. In N1-N3 group in S4A and S4B Fig, there are a total of 730 DEPs are up-regulated and 350 are down-regulated, 1355 DEGs are up-regulated and 1,170 are down-regulated. A total of 394 DEPs are up-regulated and their corresponding DEGs are unchanged, 157 DEPs are down-regulated and their corresponding DEGs are unchanged, 1,035 DEPs are unchanged and their corresponding DEGs are up-regulated, 961 DEPs are unchanged and their corresponding DEGs are down-regulated. Meanwhile, 53 DEPs and their corresponding DEGs are both down-regulated, 180 DEPs and their corresponding DEGs are both up-regulated, 140 DEPs are down-regulated and their corresponding DEGs are up-regulated, 56 DEPs are up-regulated and their corresponding DEGs are down-regulated, respectively. In N2-N3 group S4F and S4G Fig, there are a total of 345 DEPs are up-regulated and 256 are down-regulated, 1,717 DEGs are up-regulated and 2,218 are down-regulated. A total of 130 DEPs are up-regulated and their corresponding DEGs are unchanged, 81 DEPs are down-regulated and their corresponding DEGs are unchanged, 1,509 DEPs are unchanged and their corresponding DEGs are up-regulated, 2,036 DEPs are unchanged and their corresponding DEGs are down-regulated. Meanwhile, 88 DEPs and their corresponding DEGs are both down-regulated, 121 DEPs and their corresponding DEGs are both up-regulated, 87 DEPs are down-regulated and their corresponding DEGs are up-regulated, 94 DEPs are up-regulated and their corresponding DEGs are down-regulated, respectively. Additionally, the Pearson’s correlation between the proteome and transcriptome data was assessed. A positive correlation coefficient of 0.35 was found in N1-N2 group, and 0.08 in N2-N3 group, but a negative correlation between DEPs and DEGs was observed in the N1-N3 group (R = −0.03, Fig 4C, S4C and S4H Fig).

### GO and KEGG pathway enrichment analyses of DEGs-DEPs

The DEGs and DEPs were also analyzed to determine GO and KEGG pathway enrichment. GO analysis showed that DEGs-DEPs in the N1-N2 group were related to several biological processes, including cellular metabolic process, organic substance metabolic process, primary metabolic process and nitrogen compound metabolic process. The enriched cellular components included intracellular, membrane-bounded organelle and others. The molecular function of these proteins was enriched in hydrolase activity and protein binding functions (Fig 5D). However, the molecular function of DEGs-DEPs in N1-N3 group was enriched in categories related to oxidoreductase activity and hydrolase activity functions (S4D Fig). The molecular function of the DEGs-DEPs in the N2-N3 group was related to organic cyclic compound binding and heterocyclic compound binding functions (S4I Fig). KEGG pathway enrichment analysis revealed that the DEGs-DEPs of the N1-N2 group were enriched in amino sugar and nucleotide sugar metabolism, glycosphingolipid biosynthesis#x2013;globo and isoglobo series, MAPK signaling pathway, plant, starch and sucrose metabolism as well as carbon fixation in photosynthetic organisms. DEGs-DEPs in the N1-N3 comparison group were highly enriched in types of O#x2013;glycan biosynthesis, alpha–linolenic acid metabolism, glutathione, metabolism, photosynthesis, N–Glycan biosynthesis, biosynthesis of unsaturated fatty acids, phenylpropanoid biosynthesis, fatty acid degradation, various types of N–glycan, biosynthesis, arginine and proline metabolism, lipoic acid metabolism, peroxisome, valine, leucine and isoleucine degradation, sphingolipid metabolism and galactose metabolism. DEGs-DEPs in the N2-N3 comparison group were highly enriched in beta–alanine metabolism, phenylalanine metabolism, porphyrin and chlorophyll metabolism, glycine, serine and threonine metabolism, plant hormone signal transduction, cutin, suberine and wax biosynthesis (Fig 5E, S4E and S4J Fig).

### DEPs and DEGs related to drought tolerance and PPI network analysis

**Fig 6:**
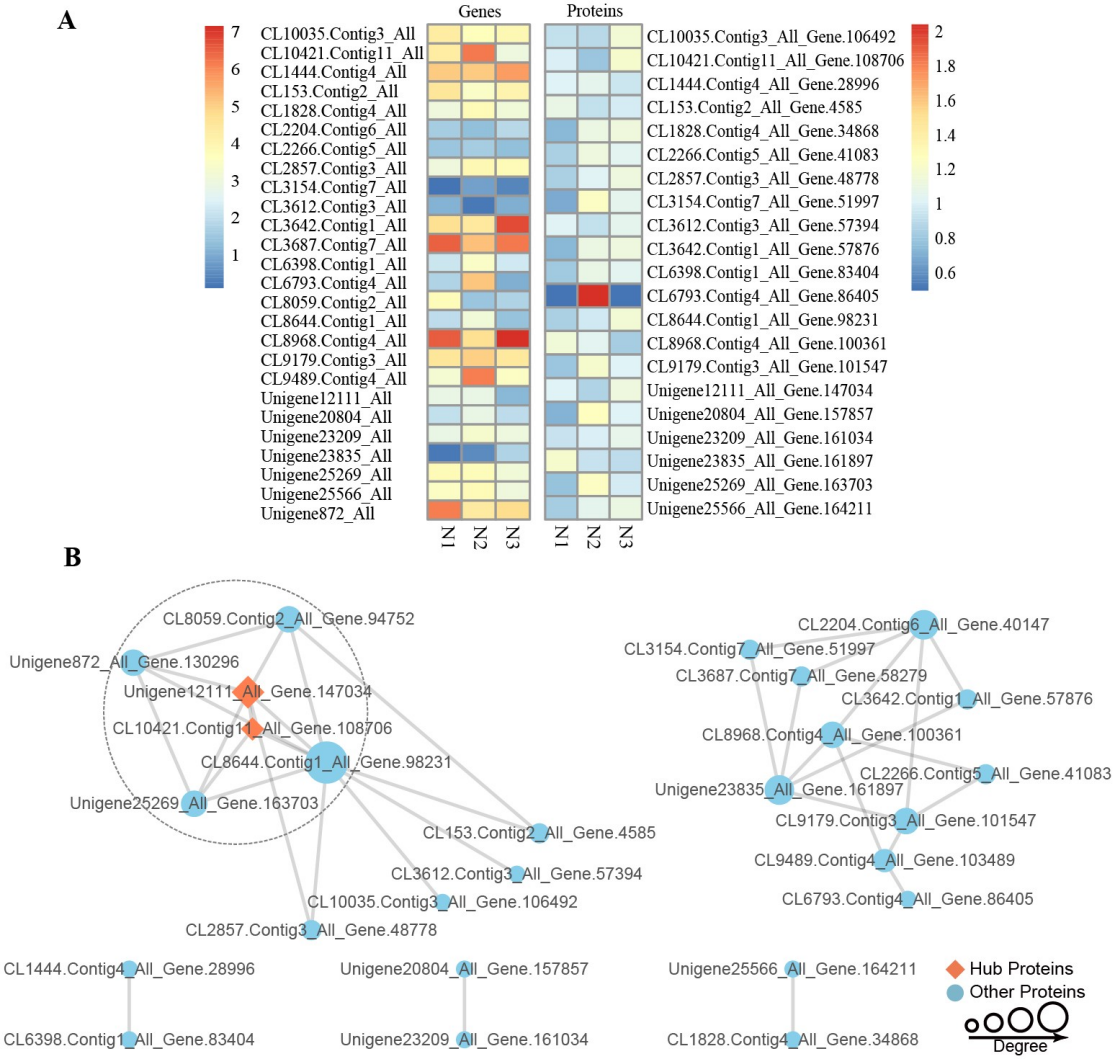
(A) Expression of drought tolerance-related DEGs-DEPs. (B) Co-expression network of drought tolerance-related DEGs-DEPs. The size of the diamond or circle represents the number of proteins that interact with a gene or protein.

To better understand the mechanism of drought tolerance in finger millet, the DEGs and DEPs with the most significant changes were further assessed. Based on a review of the literature and the GO and KEGG enrichment analyses, 113 DEGs-DEPs were found to be drought tolerance-related. Key annotations related to drought stress, including hydrolase activity, glycosyl bond activity, carbohydrate binding, hydro-lyase activity and carbohydrate binding, were assessed. In the N1-N2 group, 80 DEGs-DEPs were enriched in drought stress-related categories, including genes which had consistent and inconsistent regulation at the transcript and protein level. Within the drought treatment comparison groups, most of the DEPs and DEGs were up-regulated, implying that they may have some function in drought tolerance (Fig 6A).

The STRING database was used to predict protein interaction networks for the drought-related DEGs-DEPs, and Cytoscape was used to visualize the co-regulatory relationship between them. The resulting drought tolerance co-regulatory network consisted of 26 nodes and 36 edges (Fig 6B, S1 and S2 Tables). There were two hub genes in the network that had more than 10 edges: *BAMY2* (*CL10421*.*Contig11_All_Gene*.*108706*) and *ISA1* (*Unigene12111_All_Gene*.*147034*). Hub genes are critical nodes in regulatory networks and typically have a large variety of interaction effects. The hierarchical cluster analysis of 26 node genes showed that most had higher expression under drought stress, especially after 19 days of drought treatment (N2).

## Discussion

Plants have a variety of tolerance mechanisms to cope with biotic and abiotic stresses, including drought stress, which is a major contributor to crop productivity loss in vast areas of the world [25]. The cellular stress response pathway is controlled by many highly conserved signaling molecules and transcriptional regulators, which ultimately control gene expression. As typical C_4_ plants, cereals have strong drought tolerance and may have special proteins or regulators to integrate stress response pathways [26]. Such unique adaptations have been reported in the leaves and roots of malting barley and other cereals during drought stress response [27]. In addition, a new type of C4 model crop, foxtail millet was discovered, which has a simple and quick (CRISPR)/Cas system, providing a new method for studying C4 plants [28]. Recently, finger millet has attracted more attention due to its nutritional profile and hardiness during stress conditions [29]. In this study, drought caused finger millet to undergo significant molecular changes in order to protect itself from stress, many of which have previously been shown to take place in other drought-tolerant species [30]. A more detailed understanding of the transcriptome and proteome changes which take place in finger millet during drought stress is the key to understanding its response mechanism and identifying key regulatory genes.

This global analysis of RNA-seq and functional annotation of transcripts of finger millet leaf tissues before and after drought stress provides a comprehensive dataset of DEGs with different roles in drought stress response (Fig 2). For the drought stress and rehydration recovery comparison group, the GO and KEGG enrichment information of DEGs was analyzed. The drought-associated enriched pathways primarily related to photosynthesis, response to water deprivation, translation, ribosome process and carbon metabolism. Previous studies have also found a similar pattern in the finger millet model system during abiotic stress [31]. In the proteomics data from this study, some key protein families were found which participate in the regulation of drought stress response. This included the glycosyl hydrolase family 17 (GHL17) [32, 33] and the thaumatin family [34], which are known to play a variety of roles in plant development and response to biotic and abiotic stresses. The glutathione S–transferase (GST) and peroxidase families [35] are vital protein families that regulate active oxygen metabolism in plants. Many candidate drought-related proteins were predicted to be localized to the chloroplast and cell nucleus, indicating that they may play regulatory roles. There are also likely some new proteins to be identified to participate in the phosphorylation reaction. In plants, aquaporins (water channel proteins belonging to the major intrinsic protein (MIP) superfamily of membrane proteins) are widely present in plasma membranes and vacuolar membranes. At the protein level, certain aquaporins regulate water transport activity through phosphorylation [36]. These proteins play a role in the drought resistance of finger millet. It shows that most of the genes are transcription factors, and the drought stress of finger millet may be related to chloroplast photosynthesis [30]. Additionally, many drought-related proteins were found to be enriched in cellular metabolic process, protein turnover and hydrolase activity. Since these proteins were all up-regulated under drought, hydrolases may play a crucial role in drought tolerance in finger millet.

In order to understand the relationship between DEGs-DEPs in more detail, the identified DEGs and DEPs were subjected to co-regulation analysis. In the drought co-regulatory network, after long-term exposure to drought stress, there were more up-regulated genes related to drought than down-regulated genes, and DEGs were also up-regulated more than DEPs. By comparing the transcriptome and proteome, GO and KEGG analysis showed that drought-related DEGs-DEPs were enriched in hydrolase activity, glycosyl bond activity, oxidoreductase activity and carbohydrate metabolism.

Drought-related protein interaction networks identified 113 DEGs-DEPs that were involved in the drought stress response pathway. Two hub DEGs-DEPs were upstream of many of the drought-regulated genes and likely play regulatory roles. *BAMY2* (*barley K-amylase 2*) (*CL10421*.*Contig11_All_Gene*.*108706*) was identified as a starch debranching enzyme which inhibits dextrinase in germinating barley (*Hordeum vulgare*) [37]. Additionally, *ISA1* (isoamylase-type starch debranching enzyme) (*Unigene12111_All_Gene*.*147034*) has been shown to be involved in the biosynthesis and crystallization of starch [38]. It is thought that under drought stress, these hub genes are significantly up-regulated, thereby restricting the production of starch and dextrin in finger millet, and delaying the vegetative growth of plants to increase stress tolerance. Additionally, a stress responsive TATA-box binding protein associated factor 6 (TAF6) was found in finger millet that may also play a role in drought stress [39]. Through the use of transcriptomic and proteomic data generated from drought stress and rehydration of finger millet leaf tissues, underlying drought response mechanisms were elucidated. This led to the identification of several key transcriptional regulators and proteins which may participate in multiple different stress responses. Through additional study, these key stress response genes may be used as resources for functional analysis and genetic improvement of finger millet and other crops.

## Supporting information

S1 Fig (A) Venn diagram of differentially expressed genes between different treatment comparisons. (B) Statistics related to the number of DEGs in the transcriptome. (C) Mass spectrometry data of the proteome. (D) The number and distribution of DEPs in different comparison groups. (E) Venn diagram of transcriptome and proteome comparisons.
(PNG)

S2 Fig KOG/COG and subcellular localization prediction analysis of different drought treatment comparison groups of finger millet DEPs. (A) KOG/COG annotation of the N1-N2 DEPs group. (B) KOG/COG annotation of the N1-N3 DEPs group. (C) KOG/COG annotation of the N2-N3 DEPs group. (D) Subcellular localization prediction analysis of the N1-N2 group. (E) Subcellular localization prediction analysis of the N1-N3 group. (F) Subcellular localization prediction analysis of the N2-N3 group.
(PNG)

S3 Fig GO enrichment analysis of different drought treatment comparison groups of finger millet DEPs. (A) GO enrichment of the N1-N2 DEPs group. (B) GO enrichment of the N1-N3 DEPs group. (C) GO enrichment of the N2-N3 DEPs group.
(PNG)

S4 Fig (A) DEGs-DEPs distribution statistics in the N1-N3 DEPs group. (B) Venn diagram showing comparative analysis of DEGs-DEPs in the N1-N3 DEPs group. (C) Scatter plot between a given transcript and its one-to-one corresponding protein expression in the N1-N3 DEPs group. (D) GO enrichment analysis of the N1-N3 treatment comparison group of finger millet DEGs-DEPs. (E) KEGG pathway analysis of the N1-N3 treatment comparison group of DEGs-DEPs. (F) DEGs-DEPs distribution statistics in the N2-N3 DEPs group. (G) Venn diagram showing comparative analysis of DEGs-DEPs in the N2-N3 DEPs group. (H) Scatter plot between a given transcript and its one-to-one corresponding protein expression in the N2-N3 DEPs group. (I) GO enrichment analysis of the N2-N3 treatment comparison group of DEGs-DEPs. (J) KEGG pathway analysis of the N2-N3 treatment comparison group of DEGs-DEPs.
(PNG)

S1 Table Information about the DEGs-DEPs in the drought tolerance co-regulatory network. (XLSX)

S2 Table Candidate DEGs-DEPs which are involved in the drought stress pathway. (XLSX)
