# Supplementary figures and images for: Integration of transcriptomic and proteomic analyses for finger millet [Eleusine coracana (L.) Gaertn.] in response to drought stress
Source: PLoS One. 2021 Feb 17;16(2):e0247181. doi: 10.1371/journal.pone.0247181 (PMC7888627; doi:10.1371/journal.pone.0247181)

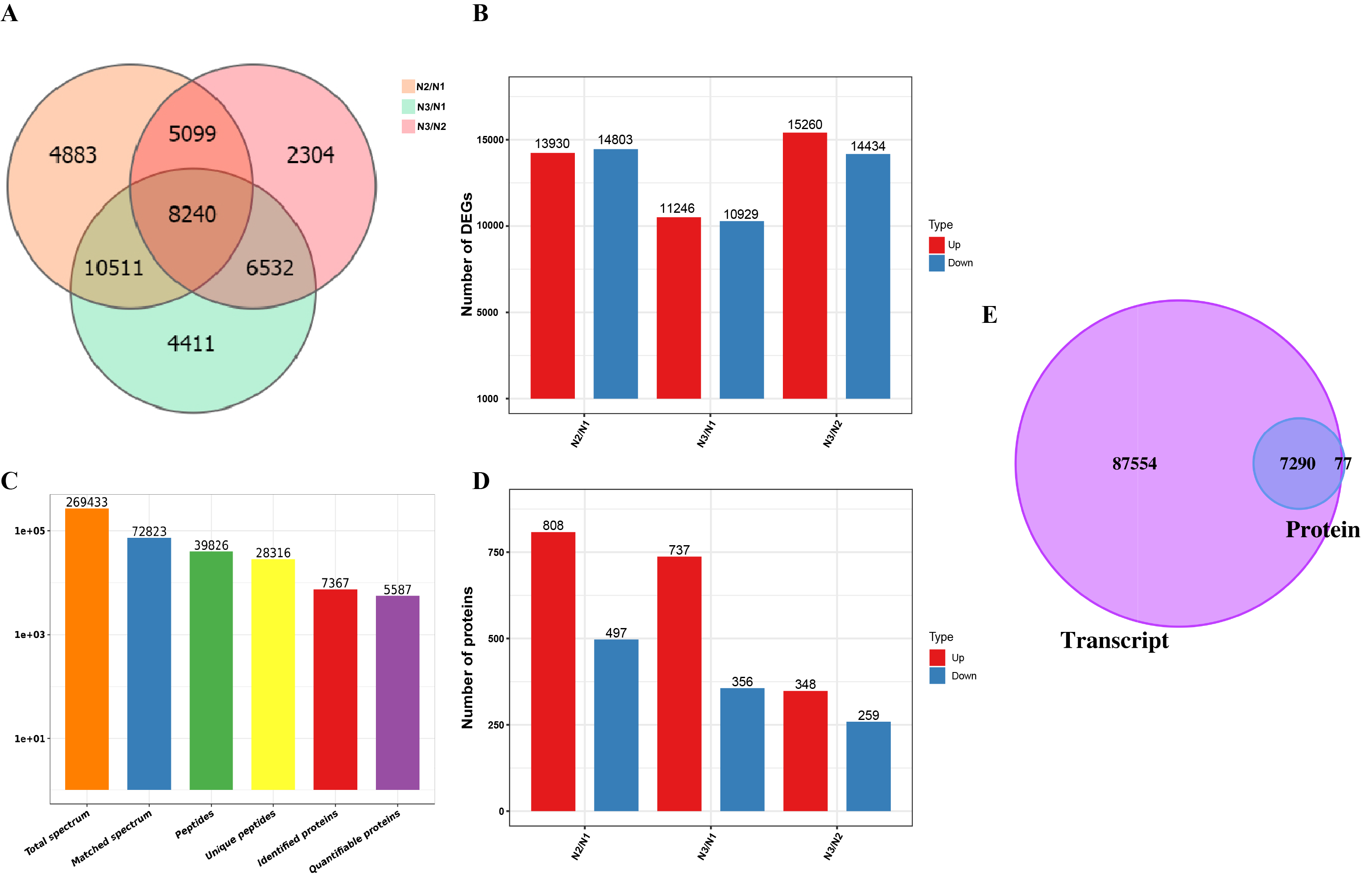

Supplement: S1 Fig — (A) Venn diagram of differentially expressed genes between different treatment comparisons. (B) Statistics related to the number of DEGs in the transcriptome. (C) Mass spectrometry data of the proteome. (D) The number and distribution of DEPs in different comparison groups. (E) Venn diagram of transcriptome and proteome comparisons. (PNG) [file pone.0247181.s001.png]

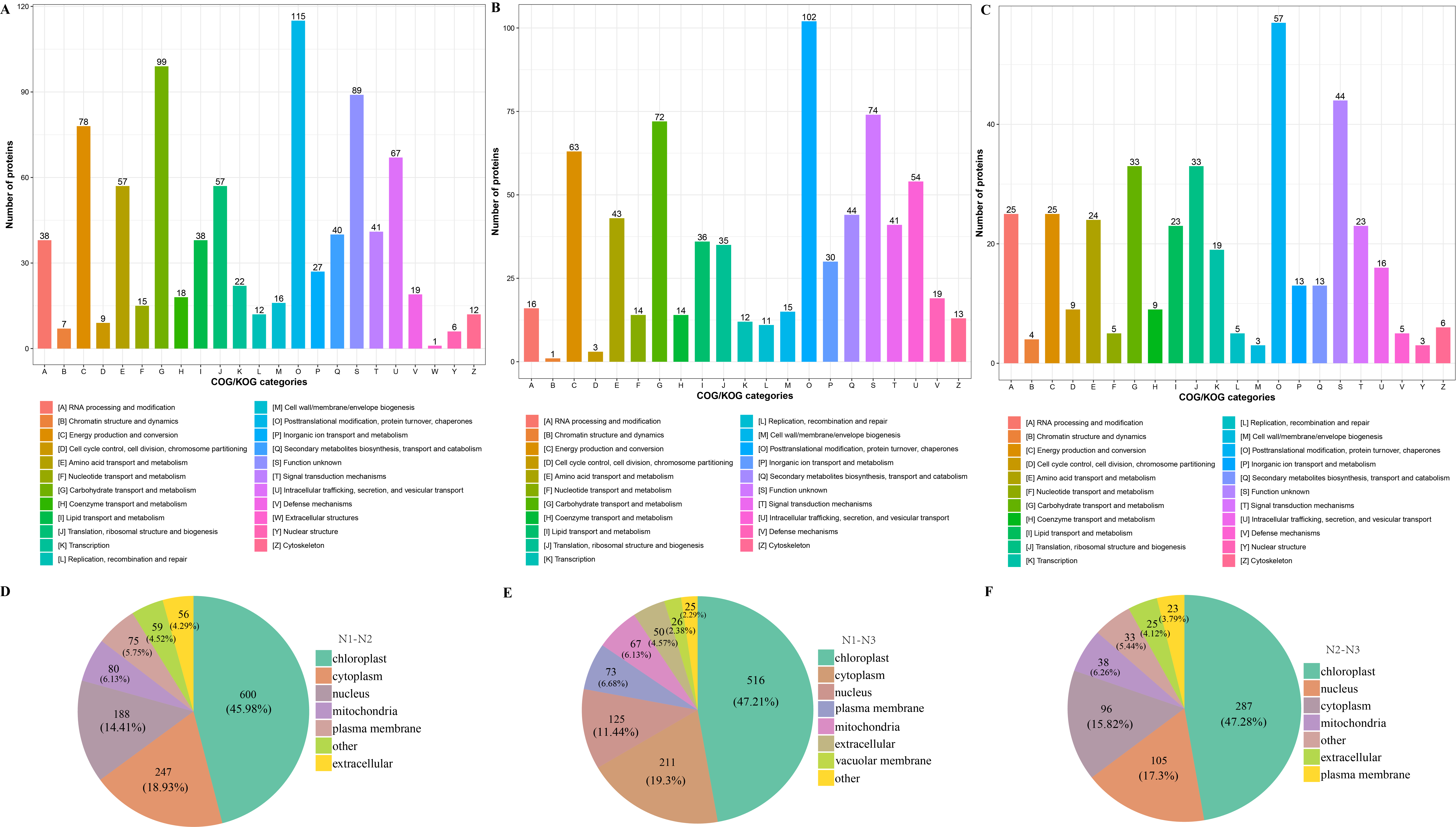

Supplement: S2 Fig — (A) KOG/COG annotation of the N1-N2 DEPs group. (B) KOG/COG annotation of the N1-N3 DEPs group. (C) KOG/COG annotation of the N2-N3 DEPs group. (D) Subcellular localization prediction analysis of the N1-N2 group. (E) Subcellular localization prediction analysis of the N1-N3 group. (F) Subcellular localization prediction analysis of the N2-N3 group. (PNG) [file pone.0247181.s002.png]

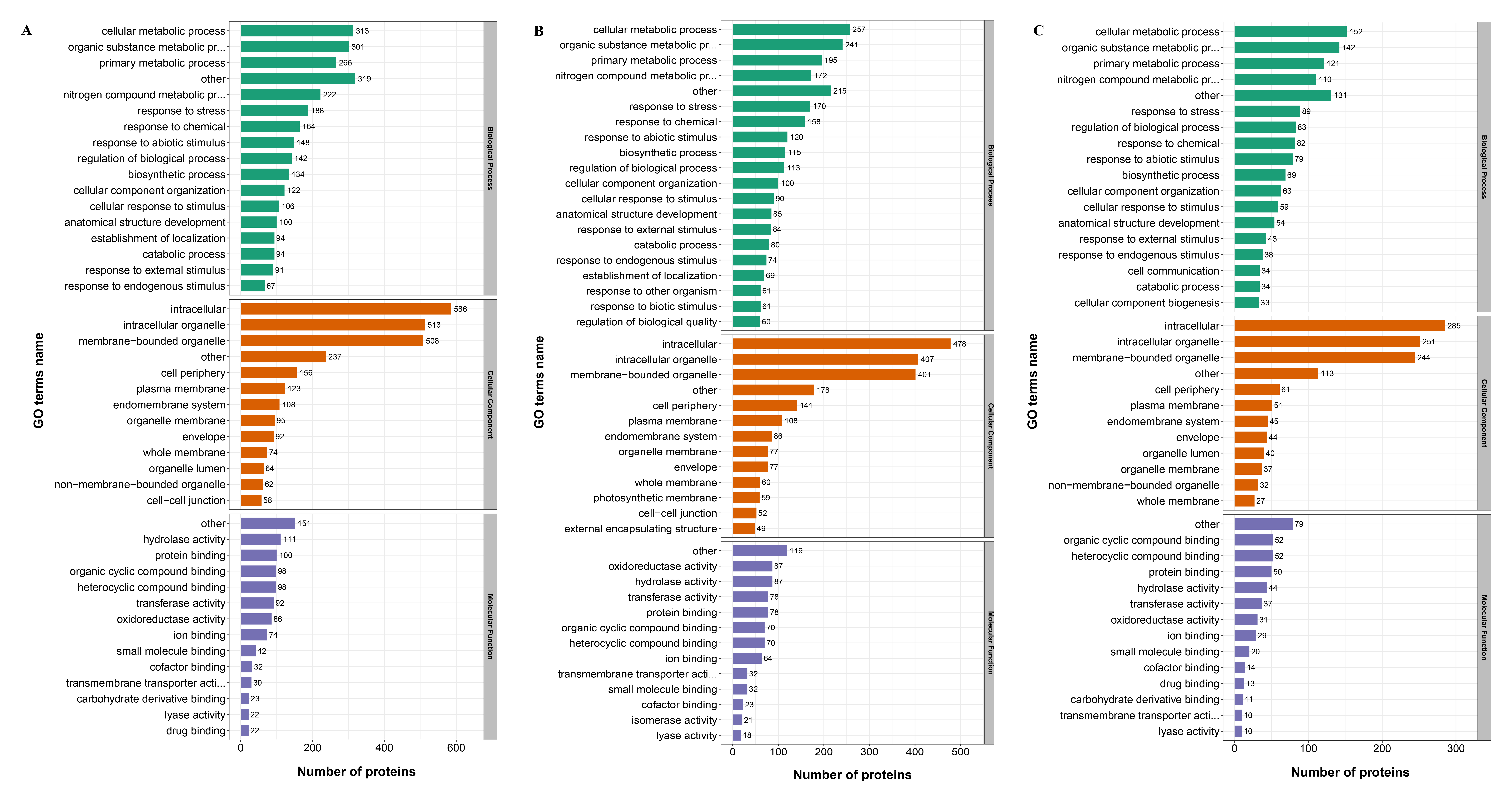

Supplement: S3 Fig — (A) GO enrichment of the N1-N2 DEPs group. (B) GO enrichment of the N1-N3 DEPs group. (C) GO enrichment of the N2-N3 DEPs group. (PNG) [file pone.0247181.s003.png]

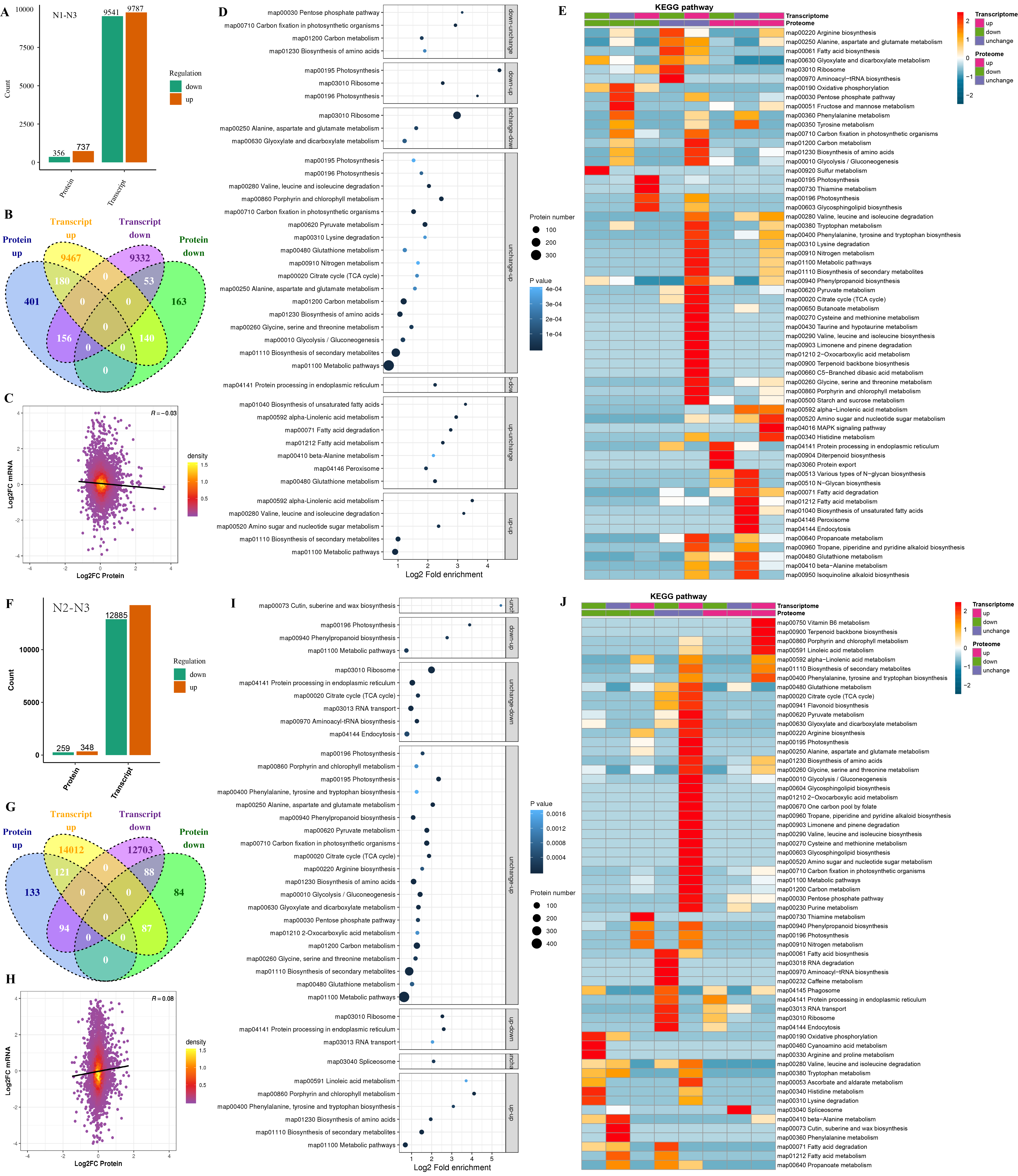

Supplement: S4 Fig — (A) DEGs-DEPs distribution statistics in the N1-N3 DEPs group. (B) Venn diagram showing comparative analysis of DEGs-DEPs in the N1-N3 DEPs group. (C) Scatter plot between a given transcript and its one-to-one corresponding protein expression in the N1-N3 DEPs group. (D) GO enrichment analysis of the N1-N3 treatment comparison group of finger millet DEGs-DEPs. (E) KEGG pathway analysis of the N1-N3 treatment comparison group of DEGs-DEPs. (F) DEGs-DEPs distribution statistics in the N2-N3 DEPs group. (G) Venn diagram showing comparative analysis of DEGs-DEPs in the N2-N3 DEPs group. (H) Scatter plot between a given transcript and its one-to-one corresponding protein expression in the N2-N3 DEPs group. (I) GO enrichment analysis of the N2-N3 treatment comparison group of DEGs-DEPs. (J) KEGG pathway analysis of the N2-N3 treatment comparison group of DEGs-DEPs. (PNG) [file pone.0247181.s004.png]
